# Changing Paradigms in Cranio-Facial Regeneration: Current and New Strategies for the Activation of Endogenous Stem Cells

**DOI:** 10.3389/fphys.2016.00062

**Published:** 2016-02-24

**Authors:** Luigi Mele, Pietro Paolo Vitiello, Virginia Tirino, Francesca Paino, Alfredo De Rosa, Davide Liccardo, Gianpaolo Papaccio, Vincenzo Desiderio

**Affiliations:** ^1^Department of Experimental Medicine, Section of Biotechnology and Medical Histology and Embryology, Second University of NaplesNaples, Italy; ^2^Medical Oncology, Dipartimento Medico-Chirurgico di Internistica Clinica e Sperimentale “F. Magrassi e A. Lanzara,” Second University of NaplesNaples, Italy; ^3^Department of Odontology and Surgery, Second University of NaplesNaples, Italy

**Keywords:** craniofacial abnormalities, regenerative medicine, stem cell transplantation, stem cells and regenerative medicine, stem cells recruitment, SDF1, bioscaffold, BMP signaling

## Abstract

Craniofacial area represent a unique district of human body characterized by a very high complexity of tissues, innervation and vascularization, and being deputed to many fundamental function such as eating, speech, expression of emotions, delivery of sensations such as taste, sight, and earing. For this reasons, tissue loss in this area following trauma or for example oncologic resection, have a tremendous impact on patients' quality of life. In the last 20 years regenerative medicine has emerged as one of the most promising approach to solve problem related to trauma, tissue loss, organ failure etc. One of the most powerful tools to be used for tissue regeneration is represented by stem cells, which have been successfully implanted in different tissue/organs with exciting results. Nevertheless, both autologous and allogeneic stem cell transplantation raise many practical and ethical concerns that make this approach very difficult to apply in clinical practice. For this reason different cell free approaches have been developed aiming to the mobilization, recruitment, and activation of endogenous stem cells into the injury site avoiding exogenous cells implant but instead stimulating patients' own stem cells to repair the lesion. To this aim many strategies have been used including functionalized bioscaffold, controlled release of stem cell chemoattractants, growth factors, BMPs, Platelet–Rich-Plasma, and other new strategies such as ultrasound wave and laser are just being proposed. Here we review all the current and new strategies used for activation and mobilization of endogenous stem cells in the regeneration of craniofacial tissue.

## Introduction

Regenerative medicine is the field of translational research that aims to replace and repair cells, tissues and organs to restore their normal functions (Mason and Dunnill, 2008). In the last 20 years, the possibility to use endogenous and exogenous stem cells for tissue repair has emerged producing enthusiasm in the scientific and medical community, but on the other side, raising a series of ethical and practical issues. The craniomaxillofacial complex is of fundamental importance for many different functions, such as breathing and eating, others non-vital but still important for social relationships, such as aesthetics and the delivery of senses such as sight, smell, and sound. For these reasons, craniomaxillofacial tissue damages have serious physiological and psychological consequences, and it is of paramount importance to seek solutions to optimize the life of people with maxillofacial trauma (Teo and Vallier, 2010). Many disciplines, such as biology, medicine, chemistry, and engineering, are involved in the study and development of techniques or products that regenerate the native conditions of such a complicated body region as the craniofacial tissues such as bone, muscle, cartilage and nervous tissue. The research moved toward two main different directions: one that involves the study of stem cell biology, including the development of novel techniques to isolate, characterize and transplant stem cells, the other related to the material science, which aims to the creation of new biomaterials and technologies suitable for the use with stem cells. As for stem cell biology, the research focused on stem and progenitor cells isolation, characterization, expansion, and transplantation for replacing damaged tissues (Giuliani et al., 2013) and on the study of growth-factors and genetic technology to control proliferation, migration and ability in tissue repair and regeneration (Alvarez et al., 2012; Han et al., 2014).

Many types of stem/progenitor cells have been described and proposed for their ability to rescue and repair injured tissue and partially restore organ function. Stem cells are undifferentiated cells capable to undergo an indefinite number of replications (self-renewal) and give rise to progenitor and finally to specialized cells. Therefore, stem cells differ from other types of cells in the body because they are capable of sustaining self-renewal, are unspecialized, and can give rise to differentiated cell types (La Noce et al., 2014a). In the field of regenerative medicine, two main types of stem cells are used: Embryonic stem cells (ESCs) and Adult stem cells (ASCs). The embryonic stem cells are isolated from the inner cell mass (ICM) of the blastocyst, they are able to differentiate in all the tissues deriving from the three definitive germ layers, and for this reason they are considered “pluripotent.” Adult stem cells reside in the adult tissues and they are pivotal for the tissue homeostasis (Mohanty et al., 2015); unlike ESCs, these cells are only able to differentiate into a limited group of cells types, and they are considered “multipotent.” In the recent years a new source of stem cells for regenerative and translational medicine has been created by transfecting adult cells (mainly dermal fibroblasts) with a varying number of stem-associated genes producing the so-called *induced Pluripotent Stem Cells* (iPSCs) (Takahashi et al., 2007). In this review, only Adult Stem Cells (ASCs) or cell-free approaches in regenerative medicine will be considered. Today, multiple sources for the isolation of adult stem cells have been identified, including heart tissue (Warejcka et al., 1996), umbilical cord blood (Campagnoli et al., 2001), skeletal muscle (Wada et al., 2002), and the dermis of skin (Toma et al., 2001). To identify a suitable cell population for craniofacial regeneration, many odontogenic stem cells, including dental pulp stem cells (Gronthos et al., 2000; Laino et al., 2005; d'Aquino et al., 2007, 2011), periodontal ligament stem cells (Seo et al., 2004), stem cells from human exfoliated deciduous teeth (Cordeiro et al., 2008), and stem cells from apical papilla (Sonoyama et al., 2008) and some non-odontogenic stem cells, including adipose-derived stem cells (Hung et al., 2011), bone marrow mesenchymal stem cells (Li et al., 2007), gingival stem cells (Hakkinen et al., 2014), embryonic stem cells (Ohazama et al., 2004), neural crest cells (Jiang et al., 2008), and even hair follicle stem cells (Wu et al., 2009) have been selected for screening. Studies have demonstrated that all odontogenic stem cells have a certain degree of multipotency *in vitro* and form pulp-dentin complexes combined with scaffold materials *in vivo*, except for PDLSCs, which tend to form bone-like tissues *in vivo* (Mangano et al., 2011; La Noce et al., 2014b; Naddeo et al., 2015). In contrast, non-odontogenic stem cells other than BMSCs have not yet been confirmed to have the potential for tooth regeneration. Among these cell types, postnatal DPSCs have the most potential as stem cells for endodontic tissue regeneration (Gronthos et al., 2002; Nakashima et al., 2004, 2009; Laino et al., 2005; Murray et al., 2007; Mangano et al., 2011; Paino et al., 2014; Naddeo et al., 2015). Nevertheless there is the need for craniofacial regeneration to repair tissue such as bone and soft tissue such as adipose tissue and among the different sources of stem cells bone marrow and adipose tissue seem to be the most promising. For what concerns material science, this constitutes one of the subjects most widely studied in recent years for its application in many fields of medicine, such as tissue engineering and regenerative medicine. The real paradigm shift that took place in the last years is the overlap of the material science with the biology, in an attempt to create new materials—also defined as *smart* materials or *bio*materials—that do not only exist as passive support to living cells, but that are also able to interact and respond sensitively to environmental cues derived from these cells (Mangano et al., 2011). Moreover, the increasing understanding of the natural mechanisms involved in the development of tissues and organs has led to the creation of three-dimensional *in vitro* bioreactors, including nanotechnology and microfluidics-based bioreactors that recapitulate normal and pathological tissue development, structure and function (Rajan et al., 2014; Obregon et al., 2015) and pave the way to the idea of “printed organ,” a new technique used for fabrication of organ-like constructs (Choi and Kim, 2015; Ledford, 2015). These technologies have been made possible thanks to the mind-shift that has brought scientists to create synthetic matrices that recapitulate the natural properties of the extracellular matrix (ECM) of the different tissues, as a direct consequence of the evidences that the composition of the scaffold is able to influence stem cell proliferation, differentiation and angiogenesis through integrin signaling (Pittenger et al., 1999; Hynes, 2002a,b).

## Grafting approaches

In last years scientific findings highlighted the importance of human cell therapy. In regenerative medicine stem cells can be used from donor that is a family member (allogeneic by family), donor unrelated (allogeneic cells from unrelated volunteer) or the patient's own (autologous). Stem cell commonly used in the therapy for craniofacial region are bone marrow stem cells(Yang et al., 2014), adipose stem cells (Griffin et al., 2014) hematopoietic stem cell (Vesterbacka et al., 2012) dental pulp stem cells (Giuliani et al., 2013). Both allogeneic and autologous cell therapies may be subdivided into two types: cell therapy for acute or for chronic conditions, the first to limit the natural progression of disease, the second for full regeneration (Sadan et al., 2008; Carpenter et al., 2009). Those approaches can be exploited in many clinical situations; therefore it is important that advantages and disadvantages of both therapies are made clear.

### Allogeneic stem cells transplant

#### Pros

Allogeneic cells represent a therapeutic technology that fits easily in pharmaceutical production. So it is more efficient to produce cells from multiple patients and introduce them into a conventional quality control (QC) system, where it is ascertained the safety of the product and its characterization from the biological point of view. As autologous cells, allogeneic cells are expandable in culture, thus allowing the production of numerous batches of material and therefore their distribution on a large scale. This represents a great potential for economic development, promoting the improvement and expansion of this therapy (Mason and Dunnill, 2008). In medical emergencies the allogeneic cells have many advantages due to the rapid availability of these cells, which guarantee a good stability during storage and already occurred characterization. It is also not necessary to perform a biopsy on a person potentially sick, avoiding additional stress to the patient and saving time.

#### Cons

The most common problem in allogeneic cell transplantation is related to the host immune response that, directed against grafted cells, can lead to strong inflammatory reaction and consequent destruction of the graft (Barker and Widner, 2004). Nevertheless, some types of stem cells as MSCs have shown immunomodulatory activity in some autoimmune disorders, such as graft-versus-host disease and systemic lupus (Sui et al., 2013; Zhao et al., 2015), but this feature seem to not be sufficient or to not apply to grafted stem cells. In fact different strategies have been proposed to overcome this problem as discussed by Guha et al. (2013). Another major problem arising with allograft is the risk of generating tumors in the host. Transplanted cells can trigger a mechanism of crosstalk with the microenvironment of the receiver, which, combined with the stem cells growth capacity, brings along the risk of uncontrolled growth after transplantation. As an example Amariglio et al. described the generation of brain benign tumor after stem cells transplantation in a patient affected by ataxia telangiectasia (Amariglio et al., 2009). This problem can also be explained by the fact that *in vitro* expanded stem cells bring increased genetic abnormalities, which can favor cancer initiation (Spits et al., 2008). In fact, before use for allogeneic transplantation, stem cells are extensively cultured, and the accumulation of genomic abnormalities may represent a risk to the recipient. Another issue in derivation and culturing stem cells for allogeneic transplantation is the use of animal products in cell culture (Foetal Bovine Serum for instance), increasing risk of graft rejection and transmission of zoonosis (Chavez et al., 2008).

### Autologous stem cells transplantation

#### Pros

The greatest advantage in the use of autologous cells is definitely to avoid immunological responses host-versus-graft. Furthermore during therapy with autologous cells you can prevent the immunosuppressive treatment on the patient, avoiding the risk of infections and lowering by far the costs of the therapy (Chen and Palmer, 2008). In bioaesthetic treatment this approach seems to be most suitable for the less intrusive therapies and look more natural given by the patient's own cells (Tsai et al., 2000). In the autograft, the cells used are isolated from the donor-patient and when the number of cells obtained by biopsy is sufficient for transplantation it is not necessary to amplify, avoiding the anomalies due to an excessive growth *in vitro* (Baker et al., 2007). In addition, they are not exposed to products of animal origin, removing from the risk of zoonosis. Finally, the patient-derived cells do not incur ethical battles and regulation, therefore their use is much simpler than that of allografts.

#### Cons

One of the major issues concerning the autograft is the limitation of material to engage in certain patients, such as children under 6 years of age or with particular diseases (Smith et al., 2011). In addition, craniofacial reconstruction may be even more difficult in the pediatric patient because the skull is not developed enough so the split-thickness bone grafting is not tolerated (Chenard et al., 2012).

Another complication is the side effects that occur in the donor site, which involves hematoma, infection, nerve damage, bone pain and fractures. All this may adversely affect the treatment and increase the length of hospital stay (Seiler et al., 2000; Smith et al., 2008). Moreover, the cells obtained by biopsy may not be sufficient for the graft, this involves an *in vitro* expansion, time consuming and often not successful. For all these reasons, this approach is not be used in emergency situations. The material used must be processed separately for each patient with customized protocols using the patient's own serum. For this reasons the use on a large scale is hardly feasible for the costs and the time required by each therapy.

## Endogenous stem cells: current approaches

The ultimate goal in the stem cell field is to find a way to translate our growing body of knowledge on stem cell biology into therapeutic applications for regenerative medicine. In that regard, most of our attention has focused upon stem-cell transplantation approaches, which have been considered above. On the other hand, the need to overcome the drawbacks associated with these approaches (mainly, the necessity of manipulating the cells before the graft takes place) has led to the development of new strategies to achieve tissue repair. The finding that many adult tissues contain stem cells that function to maintain and repair tissue damage (Su et al., 2009) paved the way to the idea that we could somehow recruit these endogenous stem cells in order to enhance tissue regeneration. Moreover, whereas the grafting strategies require the exogenous activation of stem cells, an endogenous re-activation can be speculated without the need of isolation, manipulation and grafting (Miller and Kaplan, 2012). However, though the concept of adult stem cells is an old one, the idea of their exploitation for regenerative purposes has gained attention only in the last few years. This is mainly due to the experimental evidence coming from studies showing that tissue stem cell behavior can be modulated differentially in physiologic and pathologic settings both in the animal model and in the human. For example, it is now well-established that regular exercise and brain injury can enhance neurogenesis, precursor proliferation and oligodendrogenesis in rodents (Ming and Song, 2011) and these results are comparable to those from recent imaging studies that show how aerobic exercise training enhances hippocampal volume in elderly humans (Erickson et al., 2011, 2012). Another reason we are currently approaching the idea of manipulating resident stem cells is that we now know that in some cases the factors that induce the stem cell response are the same that we have studied in other contexts, e.g., the growth factor BDNF is required for exercise-induced neurogenesis (Ming and Song, 2011). Moreover, a number of growth factors that were originally identified in stem cell culture studies have been shown to activate stem cells *in vivo* (Mitchell et al., 2004). We will now focus on the different approaches that have stemmed in regenerative medicine from the above considerations, with a special focus on craniofacial regeneration.

### Scaffolds in bone regeneration

Understanding that the three-dimensional (3D) structure of the extracellular matrix (ECM) is integral for tissue formation and regeneration has led researchers to make an effort to recreate this environment when attempting to repair tissue defects, creating biocompatible matrices known as scaffolds. A scaffold is a three-dimension biomaterial designed to allow cell-biomaterial interactions, cell survival, proliferation and differentiation. That scaffold can be designed to biodegrade at a controllable rate and is characterized by a low degree of toxicity *in vivo*. Whether produced using synthetic or biologic materials, scaffold matrices enhance tissue growth and repair by facilitating delivery and localization of progenitor cells and growth factors to a desired location (Dhandayuthapani et al., 2011). Historically, scaffolds have been used as delivery systems in cell-based applications, whereas their use in cell-free strategies for tissue regeneration is relatively new (Bueno and Glowacki, 2009).

The use of scaffolds that accurately reproduce the structure of the native ECM (the so-called *biomimetic scaffolds*) is particularly useful in cranio-facial bone defects, as they play the role of template analogous for complex anatomical form while minimizing supply constraints. These types of scaffold vary in pore size, mechanical properties and degree of biodegrability, according to their chemical structure (Teven et al., 2015; see Table 1).

**Table 1:** **Most used biomimetic scaffolds for cranio-facial bone regeneration in cell-based and cell-free applications**.

**Scaffold name**	**Chemical structure**	**Features and uses**	**Reference(s)**
**Polymer-based scaffolds**			
**NATURAL POLYMERS**			
Chitosan	Deacetylated derivative of chitin	Highly hydrophilic, osteoconductive *in vitro* and *in vivo* but not reliable for load bearing applications.	Muzzarelli et al., 1994; Seol et al., 2004
Fibroin	Insoluble protein from silk	High mechanical strength and biodegradability. Highly customizable processing (gels, sponges, nets), but lower osteoconductivity.	Riccio et al., 2012; Panda et al., 2015
Collagen	Fibrillar collagen	Highly biodegradable and osteoconductive. High osteoconductivity, but not reliable for load bearing applications.	Ferreira et al., 2012
**SYNTHETIC POLYMERS**			
Poly(lactic-co-glycolic acid) (PLGA)	Poly(α-hydroxy esters) constituted of a combination of poly(lactic acid) (PLA) and poly(glycolic acid) (PGA)	Different combinations of the two components lead to highly customizable degradation rate, mechanical properties and osteoconductivity.	Lin et al., 2002; Gentile et al., 2014; Liu et al., 2014
Poly(e-caprolactone) (PCL)	Aliphatic polyester	Long degradation time and scarce ability to mediate cellular adhesion. Useful for the production of Shape-Memory Polymers (SMPs).	Schantz et al., 2003a,b; Zhang et al., 2014
**CALCIUM PHOSPHATE-BASED CERAMIC SCAFFOLDS**			
Hydroxyapatite (HA)	Ca_10_(PO_4_)_6_(OH)_2_ (Inorganic phase of native bone)	Highly biocompatible and osteoconductive. Slow rate of degradation, affected by crystallinity. Suitable for load-bearing applications.	Yoshikawa and Myoui, 2005; Bose et al., 2012
β-tricalcium phosphate (β-TCP)	Ca_3_(PO_4_)_2_	Highly biocompatible and osteoconductive. Very brittle.	Rezwan et al., 2006; Kamitakahara et al., 2008
Amorphous Calcium Phosphate (ACP)	Ca_3n_(PO_4_)_2n_	Highly biocompatible and osteoconductive. Degradation through a process of dissolution-precipitation to form HA.	Kobayashi et al., 2014
Biphasic calcium phosphate (BCP)	Combination of HA and β-TCP	Highly biocompatible and osteoconductive. Porosity and grain size highly customizable. The addition of zinc or silicone oxide confers an increase in compressive strength and angiogenesis.	Tevlin et al., 2014
**COMPOSITE SCAFFOLDS**			
Polymer-calcium phosphate composites	Addition of Ca-P nanoparticles to polymer-based scaffolds (e.g.chitosan or fibroin)	Higher biocompatibility compared to polymer-only scaffolds and improved compressive strength compared to ceramic-only scaffolds. Superior bone growth in *in vivo* calvarial defect model.	Chesnutt et al., 2009; Qi et al., 2014
Poly(lactic acid)-calcium phosphate and poly(glycolic acid)-calcium phosphate	PLA, PGA or PLGA scaffolds added with HA	Increased compression modulus and tensile strength compared to PLGA scaffolds. Increased *de novo* bone formation.	Kim et al., 2006; Yoon et al., 2007
Poly(e-caprolactone)- calcium phosphate composites	PCL added with TCP	Less hydrophobic than PCL alone and increased compressive strength compared to TCP alone. Suitable for load-bearing applications and osteogenic differentiation of stem cells.	Liao et al., 2013; Baykan et al., 2014
Poly(1,8-octanediol-co citric acid) (POC)	Citric acid-based elastomer combined with HA and TCP	Highly biocompatible and osteoconductive. Citric acid released during polymer degradation regulates apatite nanocrystals growth, increasing stability, strength, and resistance to fracture.	Chung et al., 2011; Costello et al., 2012; Davies et al., 2014

Though a large amount of evidence correlates bioscaffold to increased tissue regeneration, it is still not clear whether this effect can be explained by the activation of endogenous stem cells (Zaky and Cancedda, 2009). For this reason, in the practice, scaffold-based approaches are generally combined with cell- and/or growth factor-based approaches. The former have already been discussed above (see section above), while the latter will be now presented.

### Growth-factors-mediated activation and/or mobilization of stem cells

**Figure 1:**
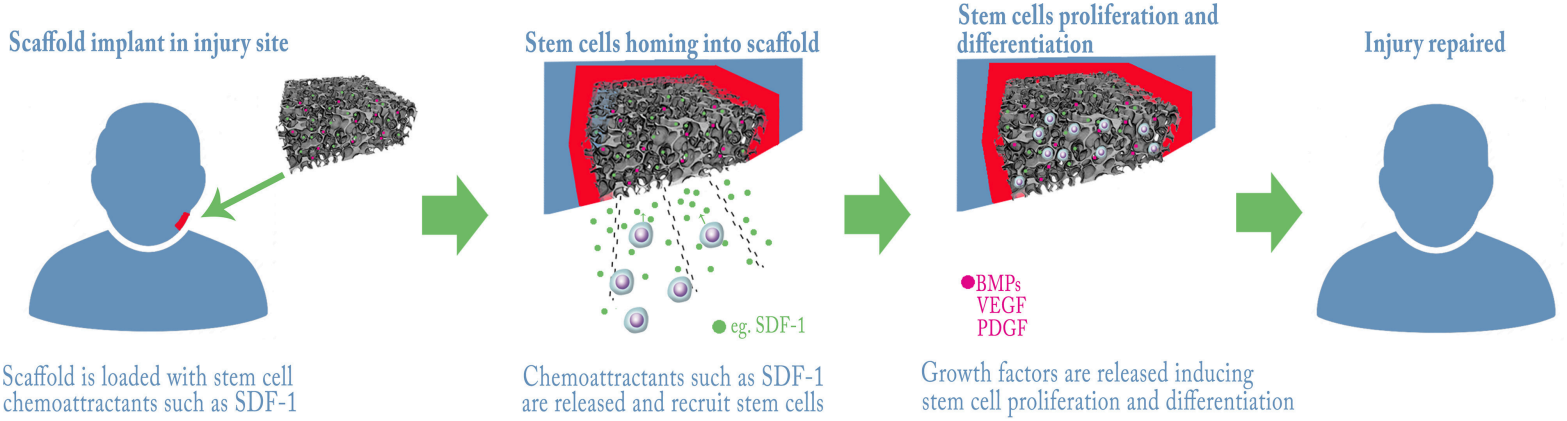
**Example strategies for bone regeneration trough Stem Cells recruitment and activation**. A scaffold with control release of chemo-attractants and/or growth factors is grafted into the lesion, endogenous stem cells are attracted and home into the scaffold where they proliferate and differentiate repairing the tissue.

Since several studies demonstrated the role of growth factors in stem cell biology (reviewed in Brizzi et al., 2012), it is almost impossible to ignore their potential provision to the therapeutic goal of tissue regeneration. Wagers previously reviewed how the use of growth factors in the clinic has already led to successful therapies in hematology and orthopedics (respectively, G-CSF analogs for chemotherapy-related neutropenia and parathyroid hormone analogs for osteoporosis; Wagers, 2012). In both these conditions, the support given to the stem cells is systemic, as it works in a tissue-specific fashion, with no privileged site of action. This is not the case in regenerative medicine, where the regeneration is usually desired only in a limited subset of body locations. For this reason, local delivery of growth factors to the site of lesion is a more suitable approach in regenerative medicine, and is usually pursued by binding growth factors to scaffolds (Figure 1). Depending on the desired release kinetic, growth factors can be either directly adsorbed to scaffolds or encapsulated in microspheres, respectively for a burst release or a sustained and delayed release. As for bone regeneration, which is the most investigated field in regenerative medicine of the craniofacial district, several growth factors have been identified with a wide range of activities *in vivo* (see Table 2). Bone morphogenetic proteins (BMPs) are a family of growth factors involved in embryonic development and regeneration of mesenchymal tissues (Zhao, 2003), whose roles have been investigated thoroughly in bone biology (reviewed in Luu et al., 2007). Although many isoforms of BMPs are known, only a small subset has been shown to be active in *in vivo* models of bone defects. BMP-2, among the others, is lethal in knockout mice model and was associated to orofacial clefting in case of haploinsufficiency in humans (Zhang and Bradley, 1996; Sahoo et al., 2011); for this reason, its use has been suggested for craniofacial bone reconstruction (Chenard et al., 2012). BMP-7 showed superior bone induction compared to autologous bone graft in an animal model of calvarial defect, when injected locally (Springer et al., 2005a,b). BMP-7 is also able to determine a widespread skeletal regeneration, and this led to its approval for use, along with BMP-2, in case of non-unions and open fractures of long bones (Gautschi et al., 2007). Other BMPs lack evidence of clinical relevance and their possible roles in the practice are reviewed by Bessa et al. (2008a,b). If, on one hand, BMPs support bone regeneration by activation of perilesional osteoprogenitor cells, several different growth factors can be used to stimulate mobilization of nearby or distant stem cells and their homing to the defect site (Herrmann et al., 2015).

**Table 2:** **Growth factors and other agents used for local activation and/or mobilization of stem cells in craniofacial and bone regeneration**.

**Agent**	**Evidence(s)**	**Reference(s)**
Bone Morphogenetic Proteins (BMPs)	Activation of mesenchymal stem cells and osteogenic differentiation. Mechanisms of action and effects vary between the 15 isoforms of BMPs. The most osteoinductive are BMP-2, -6, -9, -4, -7.	Bae et al., 2001; Zhao, 2003; Luu et al., 2007
BMP-2	Knockout mice are lethal; haploinsufficiency causes orofacial clefting in humans. Use suggested for mandible, cleft, and cranial vault reconstruction.	Zhang and Bradley, 1996; Schliephake et al., 2008; Sahoo et al., 2011; Chenard et al., 2012
BMP-4	Knockout mice are lethal; heterozygous null mice exhibit skeletal defects such as craniofacial malformations and polydactyly. Enhances bone healing when co-expressed with VEGF by genetically manipulated stem cells. May be detrimental in osseointegration of oral implants coated with collagen.	Peng et al., 2002; Zhao, 2003; Stadlinger et al., 2008
BMP-7	Knockout mice are postnatal lethal and show skeletal patterning defects in skull, hindlimbs, and ribcage. Stimulates periodontal wound healing in an animal model.	Luo et al., 1995; Giannobile et al., 1998; Springer et al., 2005a
BMP-6	Some evidences show this isoform to be one of the most osteogenic in animal models.	Solloway et al., 1998; Kang et al., 2004
Vascular Endothelial Growth Factor (VEGF)	Delivered with PLGA or collagen scaffolds enhances bone regeneration in cranium, calvarial and mandibular defects. Sustained release (up to 5 weeks) can be obtained by pre-encapsulation of VEGF in PLGA, alginate, or gelatin microspheres or, alternatively, by VEGF co-precipitation onto BCP (Basic Calcium Phosphate).	Murphy et al., 2004; Kleinheinz et al., 2005; Patel et al., 2008; De la Riva et al., 2009, 2010; Wernike et al., 2010; Behr et al., 2012; Farokhi et al., 2013, 2014
VEGF + BMP2	A combination of these two factors showed an increased vascular density during bone regeneration but no detectable enhancement in bone formation compared to BMP2 alone in animal models of cranial and mandibular defects. This combination is also capable of facilitating bone marrow stem cells (BMSCs) homing and differentiation.	Young et al., 2009; Zhang et al., 2011; Ramazanoglu et al., 2013; Zhang et al., 2014
Platelet-Derived Growth Factor (PDGF)	Local administration of PDGF in a critical-size calvarial defect has been shown to increase bone mineralization similarly to VEGF, but less than BMP-2. Use approved for periodontal repair by FDA. Chemoattractant for Mesenchymal stem cells (MSCs)	Fiedler et al., 2004; Pellegrini et al., 2009; Phipps et al., 2012; Jin and Giannobile, 2014
SDF-1	Induces extravasation and homing of mesenchymal cells through the CXCR4 receptor. SDF-1-loaded scaffolds have been studied in fracture healing models and in calvarial defects with excellent results. Capable of inducing migration, proliferation and activation of human periodontal ligament stem cells (PLSCs).	Kitaori et al., 2009; Fujio et al., 2011; Li D. et al., 2011; Du et al., 2012; Ji et al., 2013; Liu et al., 2014
SDF-1 + BMP2	The combination of SDF-1 + BMP2 increases bone volume in a calvarial defect model compared to SDF-1 alone	Jin and Giannobile, 2014
Platelet-rich Plasma (PRP)	PRP is a concentrate of blood platelets that upon activation releases various growth factors, including PDGF and VEGF. It can be used to enhance tissue healing, especially in case of concomitant cell transplantation. Potential use of PRP as a stem cell activator has been suggested in the case of periodontal regeneration.	Anitua et al., 2008; Chen et al., 2010; Del Corso et al., 2012; Fekete et al., 2012
Platelet-rich Fibrin (PRF)	PRF is a polymeric (fibrin-based) scaffold loaded with PRP that has shown good results in promoting craniofacial bone regeneration both in preclinical and in clinical setting	Simonpieri et al., 2012; Li et al., 2014

Vascular Endothelial Growth Factor (VEGF) is most known for its role in angiogenesis, but has also revealed a direct involvement in supporting bone formation (Schipani et al., 2009): for this reason, numerous preclinical bone healing models involving recombinant human VEGF delivered with various biomaterials have been tested. PLGA scaffolds delivery systems showed an increase in vascular density and bone formation in cranium and calvarial defects (Murphy et al., 2004; Kaigler et al., 2006), while collagen sponge-delivered VEGF reported similar results both in calvarial and in mandibular defects (Kleinheinz et al., 2005; Behr et al., 2012; Jin and Giannobile, 2014). Sustained release of VEGF has been achieved in different ways, such as pre-encapsulation in PLGA or gelatin microspheres or co-precipitation onto BCP, in order to support angiogenesis and bone formation for a longer period of time (Patel et al., 2008; De la Riva et al., 2009, 2010; Wernike et al., 2010; Farokhi et al., 2013, 2014). Moreover, combination of VEGF and BMP-2 showed an increased vascular density during bone regeneration but no detectable enhancement in bone formation, compared to BMP-2 alone (Young et al., 2009; Zhang et al., 2011; Ramazanoglu et al., 2013). Plateled-Derived Growth Factor (PDGF) is another angiogenetic molecule, involved in maturation of newly-formed blood vessels, but after the report of its chemoattractive action on Mesenchymal Stem Cells (MSCs) it has been studied as a homing factor in bone repair (Fiedler et al., 2004; Phipps et al., 2012). Local administration of PDGF in a critical-size calvarial defect has been shown to increase bone mineralization similarly to VEGF, but less than BMP-2 (Jin and Giannobile, 2014). Notably, Chang et al. demonstrated that gene delivery of PDGF stimulates repair of oral implant extraction socket defects in a rat model (Chang et al., 2010) moreover, PDGF has been approved for periodontal repair by FDA (Pellegrini et al., 2009). One of the most investigated signaling systems for extravasation and homing of mesenchymal cells is the SDF1-CXCR4 axis (Kitaori et al., 2009). It represents the perfect target for enhancing bone regeneration, as it is involved both in MSCs and endothelial progenitor cells (EPCs) homing dynamics, thus working both as osteoinductive and pro-angiogenetic. SDF-1-loaded scaffolds have been studied in fracture healing models (Fujio et al., 2011; Li X. et al., 2011) and in cranial defects (Ji et al., 2013; Liu et al., 2014), with excellent results (Figure 1). There is also evidence that SDF-1 may induce migration, proliferation and activation of human periodontal ligament stem cells (PLSCs; Du et al., 2012). The association of SDF-1 with BMP-2 increases bone volume in a calvarial defect model compared to SDF-1 alone (Jin and Giannobile, 2014). Another possible approach is the use of Platelet-Rich Plasma (PRP), a concentrate of blood platelets that upon activation releases various growth factors, including PDGF and VEGF (Fekete et al., 2012), and neurotransmitters that have been shown to be fundamental for tooth repair in animal model (Baudry et al., 2015). Nevertheless, its role as activator of endogenous stem cells is still controversial, this mainly due to problems in standardize the PRP production protocol (Malhotra et al., 2013); its use seems, however, more efficient in case of soft tissue healing or in case of concomitant cell transplantation (Anitua et al., 2008). In the case of periodontal regeneration, the potential use of PRP as a stem cell activator has been suggested, but not yet demonstrated (Chen et al., 2010; Del Corso et al., 2012). On the other hand, Platelet-Rich Fibrin (PRF), a polymeric (fibrin based) scaffold loaded with PRP, has shown some very interesting results in promoting craniofacial bone regeneration both in preclinical and in clinical setting (Simonpieri et al., 2012; Li et al., 2014). The use of autologous growth factors released by platelets could overcome one of the major problems associated with growth factor-mediated recruitment/activation of stem cells: the high production costs for recombinant human growth factors-loaded scaffolds. Other strategies proposed in order to stimulate the synthesis of growth factors directly at the site of the defect, such as gene transfer by plasmids, viral vectors or by genetically modified allogeneic or autologous cells, have been reviewed by Evans CH and will not be further discussed (Evans, 2014).

### Beyond growth factors

#### Small molecules and drugs

As considered in the previous section, the use of growth factors released at the site of defects to activate endogenous stem cells constitutes a valid and efficient approach for tissue regeneration, though it is encumbered by high production costs and short duration in time. If, on one hand, new preparation methods promise to decrease the costs of production, on the other hand new approaches for stem cells recruitment and activation are under investigation (Mallick and Cox, 2013). Old and new drugs are now being studied for their effects on bone and dental healing (Laurencin et al., 2014). Statins are the perfect example of drugs commonly used for hypercholesterolemia that have now shown a potential to induce bone formation (Mundy et al., 1999). The use of simvastatin-loaded scaffolds has been studied in calvarial defects in a rat model, proving an increase in bone healing; the mechanism of action seems to involve the local expression of BMP-2 and HIF-1α recruiting autogenous osteogenic and angiogenetic stem cells (Yueyi et al., 2013). AMD-3100 (octahydrochloride hydrate) is a new drug currently approved in USA (trade name: Plerixafor^TM^) for hematopoietic stem cells mobilization in patients that do not respond well to G-CSF alone. It works as a CXCR4 antagonist (or, more accurately, as a partial agonist), interfering with the SDF-1-CXCR4 axis and disrupting stem cell homing, with a primary effect on HSCs and EPCs (Pitchford et al., 2009). Several works outlined the positive effect of AMD-3100 administration after a bone lesion in animal models (Toupadakis et al., 2013) while Kumar and Ponnazhagan evidenced MSCs mobilization from bone marrow after AMD-3100 administration, and reported a significant augmentation of bone growth when it was co-administrated with IGF-1 (Kumar and Ponnazhagan, 2012). HSCs mobilization operated by G-CSF has been investigated in a phase II clinical trial in case of tibial osteotomy: in this study, the patients who received G-CSF infusion for 3 consecutive days and 4 h before surgery manifested an improved osseointegration of the bone graft compared to controls, though no difference in the quality of the newly formed bone was seen between the two groups (Marmotti et al., 2013). Local application of G-CSF with gelatin hydrogel has shown a potential use for bone regeneration as it contributes to an ideal local environment for fracture healing (Ishida et al., 2010). FTY720 is a small-molecule analog of sphingosine-1-phosphate (S1P), that works as a selective agonist on S1P1 and S1P3-5 receptors; it has proved an angiogenetic effect of microvasculature and an increase in graft osseointegration (Petrie Aronin et al., 2010; Sefcik et al., 2011). Its local delivery with PLGA-scaffolds led to statistically significant increases in bone volumes in a rat model of critical-size calvarial defects (Huang et al., 2012). Another small compound, SVAK-12, which acts as a disruptor of the binding of the E3-ligase Smurf1 with BMP-2 pathway transducers (namely, Smads -1, -5, and -8), thus increasing downstream signaling, has been found to potentiate the transdifferentiation of myoblasts toward an osteoblastic phenotype *in vitro* (Kato et al., 2011). In another study, a single local dose of SVAK-12 enhanced bone-healing in the presence of endogenous BMPs in an animal model of femoral fracture (Wong et al., 2013).

### Physical activation of endogenous stem cells

Pharmacological means are surely a powerful way to activate and/or mobilize stem cells, but other strategies are being developed that do not need the exogenous administration of substances. Though we are still at a seminal point in this direction, the idea of pursuing stem cells activation through physical means is revolutionary. Ultrasounds are mechanical waves (sound waves) characterized by frequencies higher than the upper audible limit of the human hearing. The use of ultrasounds skyrocketed in the last decades both in diagnostics and in therapy; as for bone healing, the most used ultrasound-based technique is LIPUS (Low-Intensity Pulsed Ultrasound Stimulation), which has been tested with various successes in case of bone fractures and non-unions (Watanabe et al., 2010; Yang et al., 2010). The biology of ultrasound effects on bone tissue has been a mystery until very recently, although the role of mechanical stresses is well-known in the scientific community since a long time (Carter, 1987); therefore, today is universally accepted that mechanical forces are a major determinant of stem cells self-renewal and fate decision, as their roles have been assessed in embryonic and mesenchymal stem cells (Li D. et al., 2011). In 2013, Kusuyama et al. demonstrated how LIPUS influences the multilineage differentiation of mesenchymal stem and progenitor cells, thus finding a proof of principle for ultrasound use in bone healing (Kusuyama et al., 2014). The effects of LIPUS on mesenchymal stem cells have been also studied with regards to chondrogenic differentiation (Cui et al., 2006) and osteogenic differentiation for tooth tissue engineering (Lim et al., 2013) in two separate *in vitro* studies. Light, beside the ultrasounds, is another notable mean for endogenous stem cells recruitment. In particular, in the study by Arany et al. the use of low-power lasers (LPL) induced reactive oxygen species (ROS) in a dose-dependent manner, which in turn activated latent TGF-beta via a specific methionine residue; light-activated TGF-beta was capable of differentiating human dental stem cells *in vitro*, and the results were confirmed in an *in vivo* pulp cap model in rat teeth. Moreover, in TGF-beta-RII-null animals this response to laser was abolished, confirming a central role for TGF-beta in dental regeneration (Arany et al., 2014). The last example of physical stimulus known for its effect on gene expression and stem cell fate is heat (Fan, 2012). Heat shocks constitute natural occurring events, which can be easily reproduced under specific conditions. In a series of experiments, DPSCs have been shown to be more resistant to heat stress compared to non-stem cells (Yao et al., 2011), and this paved the way for a heat-induced activation of DPSCs (Rezai Rad et al., 2013). However, these experiments were conducted *in vitro*, and their relative importance to endogenous stem cells activation has yet to be evidenced, and thus must be considered only speculative.

## Conclusions and future perspectives

Regenerative medicine is today at a crucial point: we have already developed effective methods to improve tissue regeneration for some critical districts of the human body, such as the bones of the craniofacial complex, but these methods usually require the use of cells obtained from the same individual or from another individual (respectively, autologous or allogeneic graft), that need to be processed *ex vivo* before the transplant takes place. These procedures, though effective and increasingly standardized, expose the graft recipient to the risks already discussed in the previous sections. The increasing understanding of the mechanisms that regulate stem cell biology in adult tissues and their role in tissue healing in physiological and pathological conditions, along with the better comprehension of the role of the extracellular matrix in tissue dynamics, laid the foundations to newer strategies in regenerative medicine that aim to exploit the hidden potential of endogenous stem cells, such as growth factor-loaded bioscaffolds. These new approaches have been developed in order to avoid cell processing and decrease manufacturing burden, which constitute the main limitations to the spreading of the cell-based approaches. One way to reduce consistently the production costs would be the use of gene delivery strategies, in order to induce the synthesis of the growth factors directly in the host cells. However, this approach could become detrimental, as it would suffer from the same legislative and safety concerns of gene therapy (O'Reilly et al., 2015). Another critical point is that of the almost complete lack of any kind of data on human patients for growth factors-based approaches. More clinical trials are mandatory to assess the real impact of these techniques on human health, including safety assessments for the use of known mitogens. As for drug-induced stem cell activation, on the other hand, though even more molecules are under pre-clinical investigation (Kochegarov, 2009), the ones that have already proved effective in animal models should be validated for their safety and efficacy on humans. In this context, AMD-3100 could be regarded as the ideal drug to begin the clinical investigation, as it is already approved for human use, though with a different indication. Finally, concerning the physical approaches, new rigorous studies are important to understand whether these techniques can be really considered for a clinical application, as they would constitute the easiest and the less invasive way to promote tissue regeneration.

## Author contributions

ML and VP performed the literature research and wrote the manuscript. TV and PF revised the manuscript. DA proofreading. DV and PG coordinated the work, perform the proofreading, and approve the final manuscript.

### Conflict of interest statement

The authors declare that the research was conducted in the absence of any commercial or financial relationships that could be construed as a potential conflict of interest.
